# Local Populations of *Arabidopsis thaliana* Show Clear Relationship between Photoperiodic Sensitivity of Flowering Time and Altitude

**DOI:** 10.3389/fpls.2017.01046

**Published:** 2017-06-14

**Authors:** Anna M. Lewandowska-Sabat, Siri Fjellheim, Jorunn E. Olsen, Odd A. Rognli

**Affiliations:** Department of Plant Sciences, Faculty of Biosciences, Norwegian University of Life SciencesÅs, Norway

**Keywords:** altitude, *Arabidopsis thaliana*, flowering time, local populations, photoperiodic sensitivity

## Abstract

Adaptation of plants to local conditions that vary substantially within their geographic range is essential for seasonal timing of flowering, a major determinant of plant reproductive success. This study investigates photoperiodic responses in natural populations of *Arabidopsis thaliana* from high northern latitudes and their significance for local adaptation. Thirty lineages from ten local *A. thaliana* populations, representing different locations across an altitudinal gradient (2–850 m a.s.l.) in Norway, were grown under uniform controlled conditions, and used to screen for responses to five different photoperiods. We studied relationships between variation in photoperiodic sensitivity of flowering time, altitude, and climatic factors associated with the sites of origin. We found that variation in response to photoperiod is significantly correlated with altitude and climatic variables associated with the sites of origin of the populations. Populations originating from lower altitudes showed stronger photoperiodic sensitivity than populations from higher altitudes. Our results indicate that the altitudinal climatic gradient generates clinal variation in adaptive traits in *A. thaliana*.

## Introduction

Photoperiod is an environmental cue that many organisms use to regulate seasonal changes in behavior, migration, and reproduction (Kardailsky et al., 1999; Yano et al., 2000; Bradshaw and Holzapfel, 2001; O’Malley and Banks, 2008; Zhang et al., 2008). At temperate and subarctic latitudes, photoperiod, light quality, and temperature are major environmental signals that plants sense in order to synchronize their flowering time with the changing seasons (Bradshaw et al., 2004; Putterill et al., 2004; Andres and Coupland, 2012; Song et al., 2013). Variation in flowering time of plants contributes to local adaptation to different growth conditions, and hence clinal variation in flowering time is believed to be a sign of adaptive evolution (Endler, 1977).

*Arabidopsis thaliana* is a facultative long-day plant and flowering time in response to photoperiod has been studied extensively (Lee and Amasino, 1995; Onouchi and Coupland, 1998; Simpson and Dean, 2002; El-Assal et al., 2003; Shindo et al., 2005; Balasubramanian et al., 2006; Schwartz et al., 2009; Giakountis et al., 2010). Since it is crucial for populations originating from higher northern latitudes to reproduce at a proper time of the year, one would expect that they are more sensitive to changes in photoperiod than populations from lower latitudes. However, contradictory findings have been reported. Banta et al. (2007) observed that the fitness (individual reproductive success) of accessions from Northern and Southern Europe did not differ under different photoperiods. Moreover, Samis et al. (2008) observed that photoperiodic responses in Eurasian *A. thaliana* accessions correlated with longitude, and not with latitude. However, latitudinal clines in flowering time (Stinchcombe et al., 2004; Hopkins et al., 2008), hypocotyl length in response to red and far-red light (Stenøien et al., 2002), altitudinal clines in life history traits (Montesinos-Navarro et al., 2010), and flowering time in response to vernalization have been demonstrated (Lewandowska-Sabat et al., 2012).

On a continental-wide scale, essential climatic factors vary with latitude and therefore latitudinal coordinates may be a significant proxy for climatic factors such as temperature and photoperiod. However, on smaller geographic scales, transects are present at the same latitude, e.g., due to climatic gradients related to the distance from the ocean or altitude. In these cases, latitude is not a suitable proxy for ecologically important environmental cues. Furthermore, an earlier study in our group found that altitude, and not latitude, is a better proxy for environmental cues at the northernmost range of distribution of a species, like *A. thaliana* at higher northern latitudes (Lewandowska-Sabat et al., 2012). Due to the influence of the North Atlantic Current, the temperature fluctuates during the winter season in coastal and low-altitude regions of Norway and the precipitation comes not only in the form of snow but more often in the form of rain, sleet and hail. Moderate winter temperatures, and increased winter and yearly precipitation characterize these locations. On the contrary, in regions at higher altitudes, stable low temperatures during the winter season and moderate winter and yearly precipitation result in a long lasting snow cover. In the same way as environmental cues, varying across broad geographic regions, will impose natural selection on flowering time (Engelmann and Purugganan, 2006; Hall and Willis, 2006; Verhoeven et al., 2008); we expect populations originating from restricted geographical regions, that experience extensive variation in environmental cues but also share a common demographic history, to be under selection and thus be ideal for detecting signs of local adaptation.

The photoperiod that plants experience during the growing season at higher northern latitudes depends not only on latitude of origin but also on the duration of the snow cover, determined by temperature and precipitation during autumn and winter. In the present study, we used lineages derived from high and low altitude local populations of *A. thaliana* from Norway, which represent the northernmost distribution (59–68°N) of the species. We determined photoperiodic flowering time responses by exposing plants to five different photoperiodic treatments under controlled growth conditions and scored flowering time responses, i.e., days to bolting, days to flowering, rosette leaf numbers at bolting, and flower stem length. The aim of this study was to test for relationships between variation in these flowering time responses and altitude at the site of origin of the populations, and characterize the relationship between climatic factors and variation in photoperiodic sensitivity.

## Materials and Methods

### Plant Material and Photoperiodic Response Experiment

**FIGURE 1:**
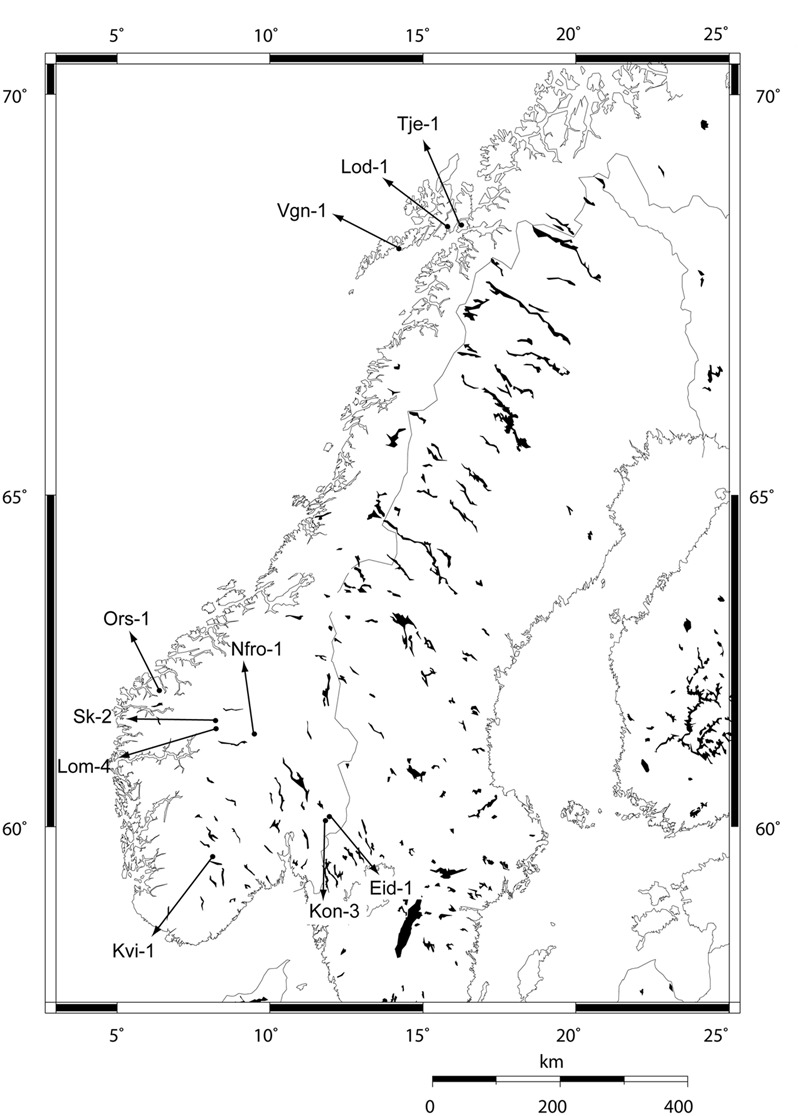
Collection sites of Norwegian *Arabidopsis thaliana* populations investigated in the study.

Ten local populations of *Arabidopsis thaliana* (L.) Heyhn, collected from high and low altitude locations in Norway, were used in this study (**Table 1** and **Figure 1**). From each site, 8–20 individuals were collected as adult plants with siliques to represent the local populations. Seeds from these individuals were propagated through a previous vernalization experiment (Lewandowska-Sabat et al., 2012). Lineages were established from the populations by single seeds picked from separate plants. Individual plants within the lineages were obtained by selfing.

**Table 1:** Collection and climate data for Norwegian populations of *Arabidopsis thaliana* used in the study.

Pop. name	Altitude (m a.s.l)	Latitude (°N)	Longitude (°E)	TJan	TJul	TYear	PJan	PJul	PYear	DL	
Ors-1	2	62°12’19”	6°32’1”	-1.0	14	6.0	199	109	2040	18.7	Western Coast/Sunnmøre
Vgn-1	3	68°10’11”	14°13’15”	-1.5	13	4.7	159	87	1500	21.7	Northern Coast/Lofoten
Lod-1	100	68°23’51”	15°56’6”	-3.1	13	3.8	158	100	1600	20.1	Northern Coast/Lofoten
Tje-1	110	68°25’29”	16°22’9”	-3.8	12	3.5	101	76	1045	23.7	Northern Coast/Lofoten
Eid-1	200	60°6’44”	12°7’28”	-6.7	15	4.2	42	82	740	17.2	Southeastern N./Kongsvinger
Kon-3	300	60°8’53”	12°5’6”	-6.7	15	4.2	42	82	740	17.2	Southeastern N./Kongsvinger
Kvi-1	703	59°29’38”	8°24’55”	-6.5	16	4.5	58	78	820	16.9	Southern N./Telemark
Sk-2	598	61°53’18”	8°18’43”	-9.4	14	2.8	25	44	317	17.0	Central N./Gudbrand Valley
Nfro-1	735	61°34’38”	9°39’42”	-11.5	15	2.4	26	60	430	16.7	Central N./Gudbrand Valley
Lom-4	850	61°40’52”	8°13’51”	-8.6	12	1.3	62	43	548	18.8	Central N./Gudbrand Valley

*Mean monthly (January: TJan, and July: TJul) and yearly (1961–1990: TYear) temperatures (°C) and precipitation (mm) (PJan, PJul, and PYear). DL, day length (h) when temperature is over 5°C and snow thawed (mean over 30 days); N., Norway.*

Screening of response to five different photoperiods (8, 16, 19, 21, and 24 h) was performed using three lineages per population and five individual plants (replicates) per lineage in each treatment. The specific photoperiods (16–24 h) were used in order to cover the full spectrum of photoperiods that the populations experience in their geographic areas of origin. The 8 h photoperiod was included as a reference short day (SD) treatment. Seeds were sown in soil (Hasselfors Garden AB, Örebro, Sweden) in pots of 6.5 cm diameter, and stratified at 4°C for 4 days in darkness to equalize the germination time. After stratification the plants were placed in a growth chamber at 23°C/8 h photoperiod with a photon flux density of 150 ± 10 μmol m^-2^ s^-1^ at 400–700 nm for 13 days in order to initiate the germination. SD was used during germination to avoid possible long-day induction of flowering already during or shortly after germination. Plants were then vernalized at 4°C under an 8 h photoperiod with a photon flux density of 50 ± 5 μmol m^-2^ s^-1^ for 14 weeks in order to saturate the vernalization requirement for all lineages completely (Lewandowska-Sabat et al., 2012) and avoid confounding effects of variation in vernalization requirement on flowering time. The light was provided by HQI lighting systems (LU400/XO/T/40 Philips General Electric, Hungary). After vernalization, the plants were transferred to five growth chambers at 16°C with the following five different day/night regimes (**Supplementary Figure S1**), i.e., (1) SD of 8 h PAR light (Photosynthetically Active Radiation)/16 h dark; (2) 16 h PAR light/8 h dark; (3) 19 h light [16 h PAR light and 1.5 h incandescent light (IL) before and after the PAR light period]/5 h dark; (4) 21 h light (16 h PAR light and 2.5 h IL before and after the PAR light period)/3 h dark; and (5) 24 h light (16 h PAR light followed by 8 h IL). The irradiance of the PAR and IL was 150 ± 10 μmol m^-2^ s^-1^ and 10 ± 2 μmol m^-2^ s^-1^, respectively, and light was provided by HQI lighting systems and incandescent bulbs (LU400/XO/T/40 Philips General Electric, Hungary and Osram, Munich, Germany, respectively). Day extension with low-intensity IL was used to avoid confounding effects due to differences in total light sum in addition to differences in day length (DL). This would have been the situation if the main day light source (HQI lamps) was also used to obtain day extensions. Furthermore, the low-intensity and lower red:far-red ratio of IL as compared to the light from HQI lamps, are more similar to the dusk and dawn conditions in nature. Flowering time was scored daily as days to bolting (DTB, date when the length of the bolting stem was approximately 1 cm), days to flowering (DTF, date of first flower to open), rosette leaf number at bolting (RLN) and flower stem length (FSL). Plants were watered every week during vernalization and twice a week during germination and exposure to the different photoperiods.

### Climate Data

Climate data for the collection sites were obtained from The Norwegian Meteorological Institute^1,2^ using data from the meteorological stations closest to the site of origin of the populations. The climate data are mean January, July, and yearly temperatures (°C) and precipitation (mm) for the period 1961–1990 (**Table 1**). Mean January and July temperatures and precipitations are the best proxies for climatic conditions in Norway, i.e., winter and summer temperature and precipitation fluctuations. Large amounts of precipitation and relatively moderate winter temperatures are characteristic for mild coastal and low-altitude sites, while moderate amounts of precipitation and low winter temperatures are associated with long winters with long lasting snow cover typical for inland and high-altitude sites. DL was calculated as the mean DL for 30 days in the spring with temperature above 5°C and no snow cover.

### Statistical Analyses

Days to bolting, days to flowering, rosette leaf number, and flower stem length data from the 8, 16, 19, 21, and 24 h photoperiod treatments were used to determine whether the 30 lineages (five individual plants of each) from 10 populations responded differently to the different photoperiods. ANOVA was used to test the main effects of photoperiod treatment and population, and the population × photoperiod treatment interaction. Significant population × photoperiod treatment interactions for days to bolting, days to flowering, rosette leaf number, and flower stem length indicate that the populations differed in their photoperiod responses. The analyses were performed using PROC ANOVA in SAS version 9.2 (SAS Institute Inc., Cary, NC, United States).

Photoperiodic sensitivity was estimated as responses to increasing photoperiods by dividing the mean values of days to bolting, days to flowering, rosette leaf number, and flower stem length at 8 h photoperiod with the respective mean values recorded at 16, 19, 21, and 24 h yielding the P16, P19, P21, and P24 photoperiodic responses (**Supplementary Table S1**). Large values indicate large photoperiodic responses, and reciprocal values were used for flower stem length for easier interpretation of the results, i.e., larger values indicating larger responses.

The overall clinal phenotypic variation associated with altitude was tested using a multivariate regression of photoperiodic responses (*n* = 16) as dependent variables, and altitude as an independent variable. The analyses were performed using the ‘MANOVA’ statement of PROC GLM in SAS version 9.2 (SAS Institute Inc., Cary, NC, United States).

We performed a canonical correlation analysis (CCA) to test the independent contribution of the traits (P16, P19, P21, and P24 for days to bolting, days to flowering, rosette leaf number, and flower stem length, respectively; *n* = 16) to the multivariate correlation with altitude, and climatic factors, respectively. We used structure correlation coefficients instead of standardized canonical coefficients because standardized canonical coefficients may be subject to multicollinearity and thus lead to incorrect interpretations (Levine, 1977). Structure correlation coefficients were used in order to compare the relative magnitudes of the effects of each trait. The magnitude of a trait’s coefficient shows the extent to which a trait correlates with a climatic gradient after accounting for a relationship between all traits (Scheiner and Gurevitch, 2001). We also used the squared canonical correlation coefficient to assess the proportion of the total variation explained by each variate. The number of variates produced in CCA is equal to the number of variables contained in the smallest matrix. Therefore, only one canonical variate was produced in the analysis with altitude, and seven in the analysis with climatic factors. The analyses were performed using PROC CANCORR in SAS version 9.2 (SAS Institute Inc., Cary, NC, United States). Correlation analyses of the structure correlation coefficients from CCA on altitude and on climatic factors were performed in Microsoft Excel. The association analyses were performed according to Montesinos-Navarro et al. (2010). Log transformed lineages means (*n* = 30) were used in the analyses.

An earlier study of Norwegian *A. thaliana* populations showed that latitude was not a good proxy for ecologically important environmental cues and geographical gradients was mainly explained by altitude in the study region (Lewandowska-Sabat et al., 2012). Moreover, the photoperiod that plants are exposed to in the growing season depends on the duration of the snow cover, which is associated with the winter temperatures and precipitation. Therefore, latitude was not included in the present analyses.

## Results

### Variation in Flowering Time

**FIGURE 2:**
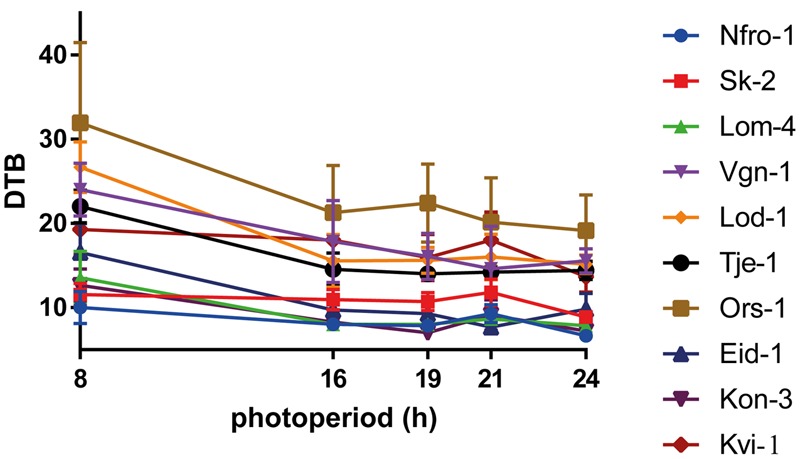
Population reaction norms of days to bolting (DTB) in response to 8, 16, 19, 21, and 24 h of photoperiod. Data are presented as population mean values ± SD (*n* = 15).

Reaction norms of days to bolting of the ten local populations are shown in **Figure 2**. In all lineages flowering was induced in SD of 8 h photoperiod. However, flowering was delayed by 2.7–12.2 days as compared to longer photoperiods and the flowering time under SD was later in low altitude compared to high altitude populations (**Figure 2**). Days to bolting, days to flowering, rosette leaf number and flower stem length were correlated with each other (data not shown). The reaction norms of days to flowering, rosette leaf number, and flower stem length are presented in the **Supplementary Figure S2**.

Significant variation in days to bolting, days to flowering, rosette leaf number, and flower stem length was found between populations in all treatments (**Figure 2** and **Supplementary Table S2**). Variance analyses showed that the effects of population, photoperiod treatment and population × photoperiod treatment interaction on days to bolting, days to flowering, rosette leaf number, and flower stem length were highly significant (*P* ≤ 0.0001; **Supplementary Table S2**). These differential responses indicate that there is genetic variation in responses to photoperiod among local populations.

### Phenotypic Variation in Photoperiod Responses Are Associated with Altitude

Multivariate regression analyses of photoperiodic response traits (*n* = 16) combined with altitude revealed that variation in photoperiod response is significantly associated with altitude (*P* ≤ 0.001). As revealed by CCA, altitude explained 87% of the multivariate trait variation (CCA; squared canonical correlation = 0.87, *P* = 0.003). CCA using climatic factors generated two significant variates that explained 98 and 94% of the multivariate trait variation, respectively (squared canonical correlation = 0.98, *P* ≤ 0.0001 for the first variate, and 0.94 and 0.007 for the second variate). We discuss only the first significant variate. The significant variate describes a climatic gradient mainly characterized by mean January temperature (-0.88), mean January (-0.91), and yearly (-0.86) precipitation, compared to mean July (0.51) and yearly (-0.69) temperatures and mean July precipitation (-0.71) and mean DL at the sites of origin in the spring with temperature above 5°C and no snow cover (-0.46; **Table 2**). Characterization of the climatic gradient is indicated by higher absolute values of the structure correlation coefficients for January temperature, and January and yearly precipitation, rather than the remaining climatic factors. Along this axis mean winter temperature, as well as mean January and yearly precipitation decreases, which is consistent with the characterization of the climatic gradient associated with altitude in our study area.

**Table 2:** Canonical correlation analyses (CCA) of phenotypic traits vs. altitude, and phenotypic traits vs. climatic factors.

	Variate	Squared canonical correlation	*F*-value	*P*-value	Structure correlation coefficient	
Phenotypic traits (*n* = 16) vs. altitude	1	0.87	5.09	0.003		
Phenotypic traits (*n* = 16) vs. climatic factors (*n* = 7)	1 2	0.98 0.93	2.78 1.95	<0.0001 0.007	TJan TJul TYear PJan PJul PYear DL	-0.88 0.51 -0.69 -0.91 -0.71 -0.86 -0.46

*Structure correlation coefficients from CCA of phenotypic traits vs. climatic factors are also presented. Mean monthly (January: TJan, and July: TJul) and yearly (1961–1990: TYear) temperatures (°C) and precipitation (mm) (PJan, PJul, and PYear). DL, day length (h) when temperature is over 5°C and snow thawed (mean over 30 days).*

**Table 3** presents the structure correlation coefficients for CCA of traits versus altitude and traits versus seven climatic factors. The magnitude and signs of the structure correlation coefficients for each trait are similar for all CCAs. Coefficients from CCA on altitude and on climatic factors are highly correlated (0.87). Bolting and flowering time (days to bolting and days to flowering) in response to longer photoperiods (16, 19, 21, and 24 h, compared to 8 h) were negatively correlated with altitude (**Table 3**; except for P24_DTF), i.e., bolting and flowering in response to long photoperiods were more rapid in populations from lower altitudes. Responses of rosette leaf number and flower stem length to longer photoperiods were positively correlated with altitude (**Table 3**; except for P24_FSL), i.e., in populations from higher altitudes there were fewer rosette leaves and longer flower stem produced in response to longer photoperiods compared to 8 h photoperiod. The highest absolute values of the structure correlation coefficients were found for the responses to 21 h photoperiod versus altitude (**Table 3**). Flowering in response to 21 h photoperiod (P21_DTF) versus altitude are presented in **Supplementary Figure S3** (structure correlation coefficient = -0.47). These analyses confirm that climatic gradients associated with altitude explain a large proportion of variation in photoperiodic response.

**Table 3:** Structure correlation coefficients for canonical correlation analyses (CCA) of traits vs. altitude and traits vs. seven climatic factors.

	Altitude	Climatic factors
P16_DTB	-0.2254	-0.1599
P19_DTB	-0.1519	-0.0691
P21_DTB	-0.4558	-0.2894
P24_DTB	-0.2000	-0.2005
P16_DTF	-0.1460	0.002
P19_DTF	-0.0351	0.1144
P21_DTF	-0.4725	-0.2564
P24_DTF	0.1144	0.2008
P16_RLN	0.2632	0.4797
P19_RLN	0.1542	0.2802
P21_RLN	0.3030	0.4604
P24_RLN	0.1631	0.3265
P16_FSL	0.3455	0.2333
P19_FSL	0.4020	0.2662
P21_FSL	0.4780	0.4174
P24_FSL	-0.0809	-0.3828
Correlation of coefficients from altitude vs. climatic factors CCA		0.87

*Only the first variate from the climatic factors CCA is presented in the table. P, photoperiodic response to 16, 19, 21, and 24 h of treatment, respectively, compared to 8 h. DTB, days to bolting; DTF, days to flowering; RLN, rosette leaf number; FSL, flower stem length.*

## Discussion

We have demonstrated that photoperiodic responses in Norwegian populations of *A. thaliana* originating from its northernmost distribution range were associated with altitude and several climatic factors (**Table 3** and **Supplementary Figure S3**). It is therefore highly likely that climatic factors that are changing with altitude, e.g., winter mean temperatures and precipitation, can impose selection pressures on photoperiodic responses in *A. thaliana*. All of the populations flowered in SD, which is due to the vernalization treatment they were subjected to prior the exposure to five different photoperiodic treatments. The vernalization treatment of the populations were applied in order to fully saturate their vernalization requirements, as the large majority of the populations did not flower without a prolonged cold treatment requiring 6–9 weeks to fully saturate their vernalization requirement (Lewandowska-Sabat et al., 2012). Despite the fact that the vernalization requirement was fully saturated for all populations, flowering was markedly promoted by increasing the photoperiod from 8 to 16 h (i.e., earlier flowering at 16 compared to 8 h; **Figure 2**), which indicates that the critical photoperiod for flowering in these populations is between 8 and 16 h (Roberts and Summerfield, 1987; Pouteau et al., 2008; Giakountis et al., 2010).

Although photoperiod is highly associated with latitude of origin, many other environmental factors, not associated with latitude also affect flowering time, survival, and seed production. Consequently, differences in temperature, precipitation, and duration of snow cover during the winter season may create different selective pressure on photoperiodic control of flowering. Longer photoperiods enhanced flowering in populations from lower altitudes (**Table 3**), and these populations demonstrated lower vernalization requirements in our previous study (Lewandowska-Sabat et al., 2012). Thus, it seems likely that natural selection at low altitude locations are creating populations which are more photoperiodic sensitive than at higher altitudes since, at lower altitudes, photoperiod is a more reliable indicator of the changing seasons than temperature. Populations from low altitudes might perceive longer periods with increasing photoperiod as signal for the onset of spring. This gives insurance against damages to floral organs caused by sudden frost in early spring (Tonsor and Scheiner, 2007).

Furthermore, the highest absolute values of the structure correlation coefficients were found for responses of all populations to 21 h photoperiod (**Table 3**). This indicates the highest association of responses to this photoperiod with altitude, which may indicate that these populations are adapted to photoperiods of approximately 21 h.

In low altitude regions, where winters are relatively mild and short, and with unpredictable temperature fluctuations, it is likely that plants respond rapidly to increasing photoperiod in spring, the crucial signal for these populations to flower at the right time. On the contrary, in high altitude locations, where winters are harsh with long lasting snow cover, rapidly increasing temperatures in the spring are more reliable signals than changing photoperiod, for these populations to flower.

In high altitude populations, the enhanced rosette leaf number responses to photoperiod were observed (**Table 3**). The greater rosette leaf number responses indicate reduction in the leaf number, i.e., fewer rosette leaves were produced in response to longer photoperiods compared to 8 h. This may suggest that at higher altitudes plants with fewer rosette leaves are favored by natural selection because they likely overwinter under snow cover as rosettes and are adapted to water loss during winter months. These is consistent with previous findings of a genetic correlation between water use efficiency and ecological traits (e.g., rosette leaf number) in *A. thaliana* (McKay et al., 2003; Stinchcombe et al., 2004). Furthermore, trends of smaller plant size (fewer leaves) at high altitudes were observed in *A. thaliana* (Luo et al., 2015), and it has been suggested that this may be a mechanism that allow plants to benefit from higher temperatures closer to the soil (Körner, 1999).

We have observed that in low altitude populations, flower stem length responses to longer photoperiods were decreased (**Table 3**), i.e., these populations developed shorter flower stem in response to longer photoperiods compared to high altitude populations. Flower stem elongation in *A. thaliana* is regulated by gibberellin, an endogenous regulator of plant growth and development (Richards et al., 2001). It has been shown that treatment with inhibitors of gibberellin biosynthesis suppresses stem elongation under long day conditions in spinach (Zeevaart et al., 1993). Also, in *A. thaliana* and pea (*Pisum sativum*), lower day temperature was shown to decrease gibberellin levels through enhanced gibberellin inactivation, as compared to higher temperature (Stavang et al., 2005, 2009). Thus, it is likely that endogenous gibberellin regulation play an important role in inhibition of stem elongation in low altitude populations, preventing them from too rapid growth in unfavorable environmental conditions, such as in cold period of early spring.

In order to reflect the natural growth conditions in Norway, we used a much lower ambient temperature (16°C) than the standard temperature (21°C) used in most of the *A. thaliana* studies (Hoffmann, 2002). Therefore, interacting effects of photoperiod and lower ambient temperature (Halliday et al., 2003), as being used in our study, have the potential to reveal phenotypic responses involving genetic regulations that so far has gone undetected in *A. thaliana*. On the other hand, these conditions may still be artificial, especially in the autumn when SDs are often accompanied by very cold weather. Furthermore, in order to test whether the genetic differences in photoperiod sensitivity observed in our study are under natural selection, a comparison of results from common garden experiments at low- versus high altitude locations could provide further evidences.

## Conclusion

We have shown that photoperiodic sensitivity of *A. thaliana* populations originating from its northernmost species boundary is associated with altitude. Populations from lower altitudes were more responsive toward changes in photoperiod than populations from higher altitudes. Therefore, it is highly likely that climatic factors such as length of the winter and duration of snow cover may impose natural selection that creates clinal variation in photoperiodic sensitivity in *A. thaliana*. These results indicate that higher photoperiodic sensitivity in lower altitude populations might be essential for sensing the onset of spring in regions with relatively mild and unpredictable winter climates as opposed to higher altitude climates with more stable winters. However, this needs to be verified by further studies. Nevertheless, this study contributes to the current knowledge on diversity and dynamics of photoperiodic responses in natural populations of *A. thaliana*.

## Author Contributions

AL-S participated in the design of the study, carried out the experiments, collected and analyzed the data, and drafted the manuscript. SF, JO, and OR participated in the design of the study, discussion and interpretation of the results, and manuscript drafting. All authors read and approved the final version of the manuscript.

## Conflict of Interest Statement

The authors declare that the research was conducted in the absence of any commercial or financial relationships that could be construed as a potential conflict of interest.

## Footnotes

1 https://www.met.no/en

2 http://www.senorge.no/aboutSeNorge.html

## Supplementary Material

FIGURE S1 Set up of the day and night regimes during the experiment. Populations of *Arabidopsis thaliana* were screened for responses to 8, 16, 19, 21, and 24 h of photoperiod.

FIGURE S2 Population reaction norms of **(A)** days to flowering (DTF), **(B)** rosette leaf numbers at bolting (RLN), and **(C)** flower stem length (FSL) in response to 8, 16, 19, 21, and 24 h of photoperiod. Data are presented as population mean values ± SD (*n* = 15).

FIGURE S3 Scatter plot of the mean lineage values (three lineages per population were used with five replicates per lineage) for response to 21 h of photoperiod (days to flowering DTF) vs. altitude of origin.

TABLE S1 Phenotypic data for lineages from 10 populations of *Arabidopsis thaliana* used in the study. P, photoperiodic response to 16, 19, 21, and 24 h of treatment, respectively, compared to 8 h. DTB, days to bolting; DTF, days to flowering; RLN, rosette leave number; FSL, flower stem length.

TABLE S2 ANOVA of population, photoperiod treatment and population × photoperiod treatment interaction effects on days to bolting (DTB), days to flowering (DTF), rosette leaves number (RLN), and flower stem length (FSL). Population and photoperiod treatment effects were tested against the population × photoperiod treatment interaction effect.
