# Supplementary figures and images for: Local Populations of Arabidopsis thaliana Show Clear Relationship between Photoperiodic Sensitivity of Flowering Time and Altitude
Source: Front Plant Sci. 2017 Jun 14;8:1046. doi: 10.3389/fpls.2017.01046 (PMC5469908; doi:10.3389/fpls.2017.01046)

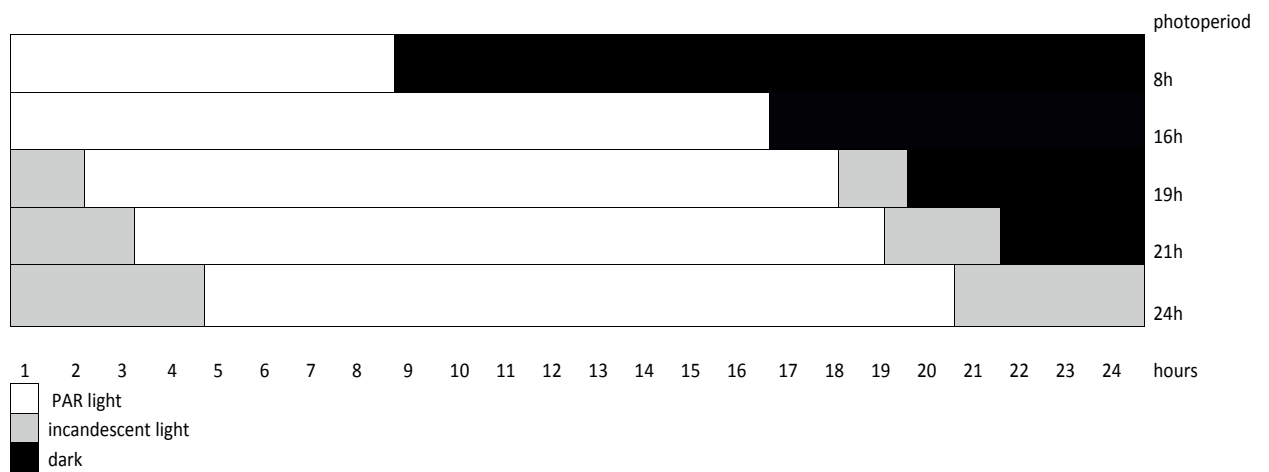

Supplement: FIGURE S1 — Set up of the day and night regimes during the experiment. Populations of Arabidopsis thaliana were screened for responses to 8, 16, 19, 21, and 24 h of photoperiod. [file Image_1.PDF]

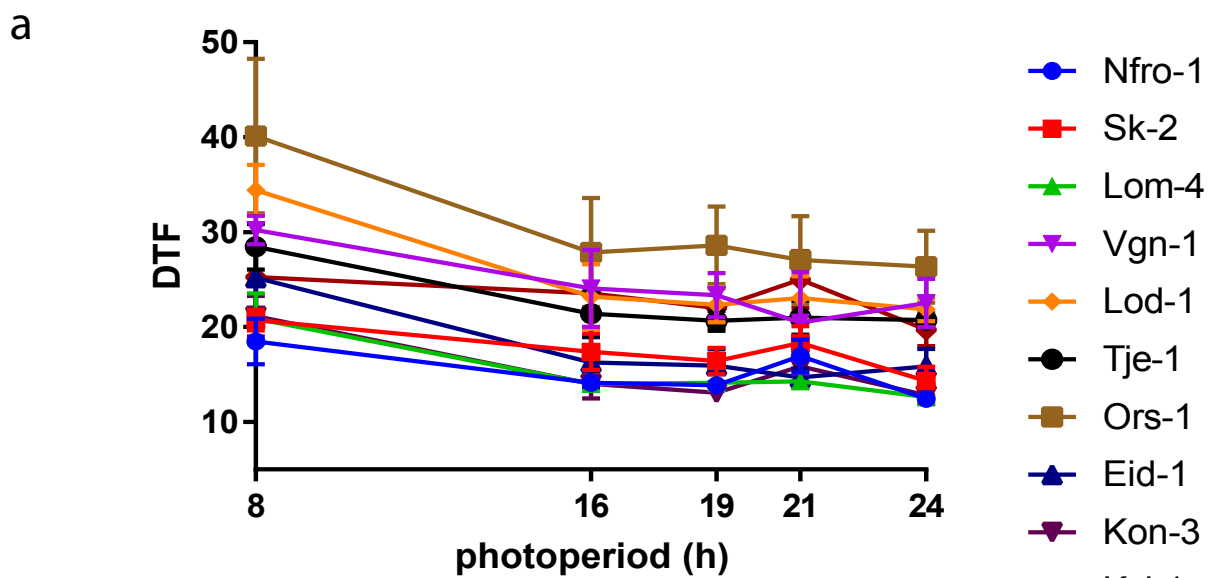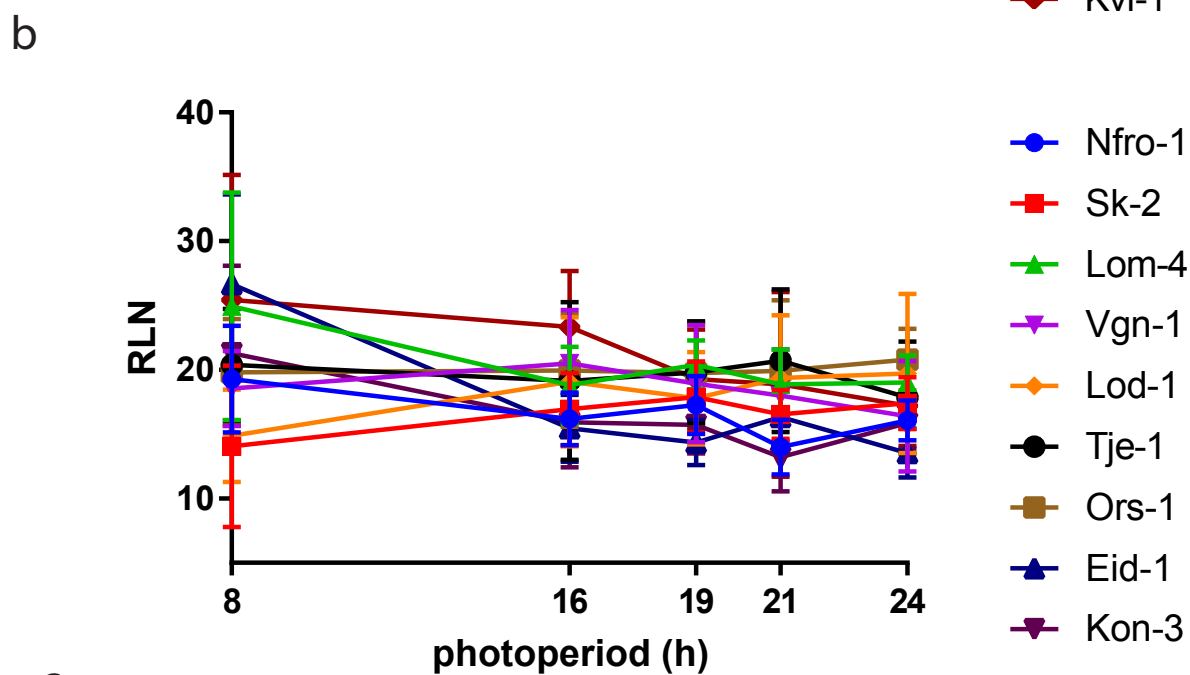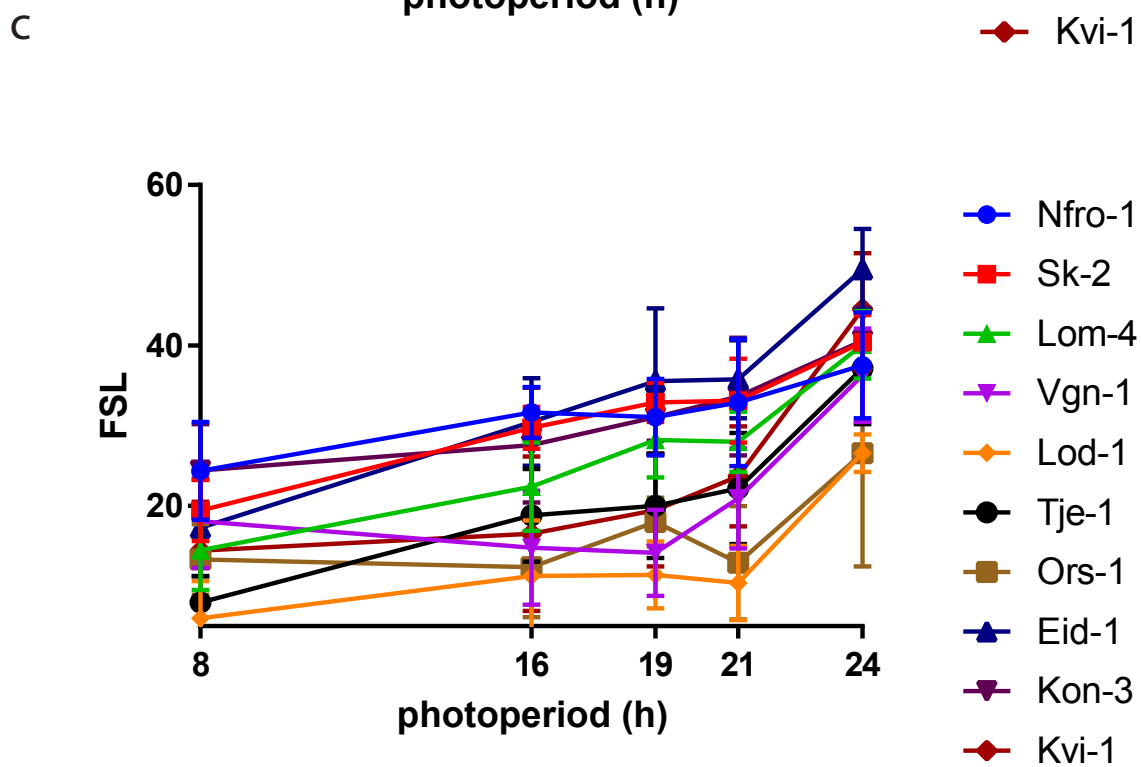

Supplement: FIGURE S2 — Population reaction norms of (A) days to flowering (DTF), (B) rosette leaf numbers at bolting (RLN), and (C) flower stem length (FSL) in response to 8, 16, 19, 21, and 24 h of photoperiod. Data are presented as population mean values ± SD (n = 15). [file Image_2.PDF]

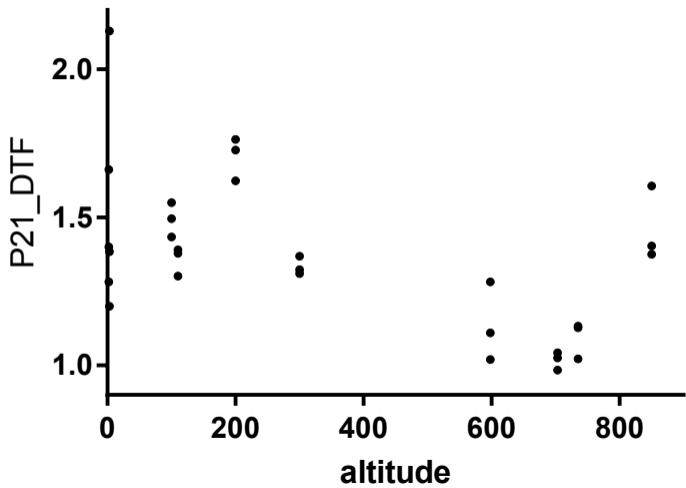

Supplement: FIGURE S3 — Scatter plot of the mean lineage values (three lineages per population were used with five replicates per lineage) for response to 21 h of photoperiod (days to flowering DTF) vs. altitude of origin. [file Image_3.PDF]
