# Supplementary material for: Local Populations of Arabidopsis thaliana Show Clear Relationship between Photoperiodic Sensitivity of Flowering Time and Altitude
Source: Front Plant Sci. 2017 Jun 14;8:1046. doi: 10.3389/fpls.2017.01046 (PMC5469908; doi:10.3389/fpls.2017.01046)
Supplement: TABLE S2 — ANOVA of population, photoperiod treatment and population × photoperiod treatment interaction effects on days to bolting (DTB), days to flowering (DTF), rosette leaves number (RLN), and flower stem length (FSL). Population and photoperiod treatment effects were tested against the population × photoperiod treatment interaction effect. [file Table_2.DOCX]

Table S2. ANOVA of population, photoperiod treatment and population x photoperiod treatment interaction effects on days to bolting (DTB), days to flowering (DTF), rosette leaf number (RLN) and flower stem length (FSL). Population and photoperiod treatment effects were tested against the population x photoperiod treatment interaction effect.

| Effects |  | DTB | | | DTF | | RLN | | FSL | |
| --- | --- | --- | --- | --- | --- | --- | --- | --- | --- | --- |
|  | df | | MS | F-value | MS | F-value | MS | F-value | MS | F-value |
| Population | 9 | | 1807.8 | 231.28^***^ | 1895.6 | 258.78^***^ | 212.0 | 11.45^***^ | 3520.0 | 102.24^***^ |
| Phot. treat. | 4 | | 1152.2 | 147.41^***^ | 1700.3 | 232.12^***^ | 231.3 | 12.49^***^ | 9359.8 | 271.85^***^ |
| Pop x phot.tr. | 36 | | 49.2 | 6.3^***^ | 45.4 | 6.21^***^ | 87.8 | 4.74^***^ | 200.5 | 5.82^***^ |

******* p-value ≤ 0.0001
